# Depression, Anxiety, Stress and Distress Following Cytoreductive Surgery and Hyperthermic Intraperitoneal Chemotherapy: Results of a Prospective Cohort Study

**DOI:** 10.1007/s10880-022-09918-0

**Published:** 2022-11-07

**Authors:** Amy Oswald, Kate McBride, Susan Seif, Cherry Koh, Nabila Ansari, Daniel Steffens

**Affiliations:** 1 Psychology Department, Royal Prince Alfred Hospital, Sydney, NSW Australia; 2 Surgical Outcomes Research Centre (SOuRCe), Royal Prince Alfred Hospital, Sydney, NSW Australia; 3 Faculty of Medicine and Health, Central Clinical School, The University of Sydney, Sydney, NSW Australia; 4 RPA Institute of Academic Surgery (IAS), Royal Prince Alfred Hospital and The University of Sydney, Sydney, NSW Australia

**Keywords:** Depression, Anxiety, Stress, Distress, Surgical Outcomes

## Abstract

The aim of this study was to describe the levels of depression, anxiety, stress, and distress in patients undergoing cytoreductive surgery (CRS) and hyperthermic intraperitoneal chemotherapy (HIPEC). The 21-item Depression, Anxiety and Stress Scale (DASS-21) and Distress Thermometer were administered preoperatively, postoperatively day 10, and at hospital discharge to 169 patients with peritoneal carcinomatosis undergoing CRS and HIPEC. The mean preoperative values for DASS-21 subscale scores were 4.7 (depression), 4.2 (anxiety), and 8.4 (stress), and the mean preoperative Distress Thermometer rating was 4.0. No significant changes in levels of depression, stress, or distress were noted thereafter. The DASS-21 anxiety subscale score significantly increased at hospital discharge (*p *= .005). Higher levels of preoperative psychological depression, anxiety, stress and distress were associated with worse mental component scores. Higher preoperative depression levels were associated with the provision of more clinical psychologist occasions of service, and higher preoperative distress levels were associated with younger age. Preoperative psychological measures are important for ensuring CRS and HIPEC patients that require additional support are identified and provided with ongoing psychological interventions.

## Introduction

The treatment of patients presenting with peritoneal carcinomatosis, including metastatic cancers arising from the colon, rectum, appendix, ovary and mesothelium, remains a challenge. Recently, the combination of cytoreductive surgery (CRS) and hyperthermic intraperitoneal chemotherapy (HIPEC) are associated with increased 5-year survival rates when compared to other treatment options, such as systemic chemotherapy or palliative care.(Ansari et al., 2019). CRS is a radical surgical procedure that aims to remove all visible tumour deposits from the peritoneal cavity. HIPEC is the intraoperative administration of heated chemotherapy agents in the intraperitoneal cavity following CRS, to eradicate any residual microscopic disease (Klaver et al., 2012). This is a complex and aggressive surgical treatment that offers the prospect of extended survival at the risk of considerable morbidity and mortality (Glockzin et al., 2009). The postoperative morbidity associated with CRS and HIPEC may include respiratory complications, prolonged bowel obstruction, wound collection, pain and sepsis.

There is a paucity of evidence surrounding the postoperative psychological pathway and influence on in-hospital surgical outcomes for patient following CRS and HIPEC. A recent study examining correlations between distress scores and clinical outcomes in those receiving this treatment found no link to the patient’s complication rate (Brandl et al., 2019). However, this study measured distress only preoperatively. To our knowledge no studies have reported on the longitudinal course of psychological variables within the length of hospital stay following CRS and HIPEC.

More long-term studies of patients’ self-reported mental health after CRS and HIPEC suggests a stable or improving trajectory of distress. A prospective cohort study demonstrated the maintenance of a mental component score from preoperatively to 12 months after surgery (Steffens et al., 2020), whereas Dodson et al. found that mood symptoms after CRS and HIPEC improved 6 and 12 months postoperatively (Dodson et al., 2016). Further, Chia et al. (Chia et al., 2016) reported improvement in anxiety at six months after CRS and HIPEC.

There is growing evidence suggesting that patients presenting with preoperative psychological distress are more likely to have worst postoperative surgical outcomes (Connerney et al., 2001; Fox et al., 2013; Mavros et al., 2011; McBride et al., 2018; Tully et al., 2008). A recent systematic review and meta-analysis, including over 6 million patients, demonstrated that patients presenting with preoperative mental illness were more likely to experience poorer surgical outcomes such as presence of postoperative complications and longer length of hospital stay (McBride et al., 2021). However, none of these published studies included patients undergoing surgery for peritoneal carcinomatosis. Thus, the association between preoperative psychological status and postoperative surgical outcomes during hospitalisation for CRS and HIPEC patients is not known.

The primary aim of this study was to describe the self-reported levels of depression, anxiety, stress, and distress in patients with peritoneal carcinomatosis undergoing CRS and HIPEC before and after surgery. The secondary aim was to measure the associations between preoperative self-reported depression, anxiety, stress, and distress and patient characteristics, postoperative surgical outcomes, and quality of life.

## Methods

### Study Population

This study was an observational prospective cohort study of adult patients (aged ≥ 18 years) from Royal Prince Alfred Hospital in Sydney, Australia who underwent elective CRS and HIPEC for peritoneal carcinomatosis between April 2017 and April 2019. This study was approved by the Sydney Local Health District Human Research Ethics Committee (Approval number X18-0531).

### Model of Care

Patients undergoing maximally invasive CRS and HIPEC are seen by an extensive multi-disciplinary team, including specialist colorectal surgeons, senior medical oncology, anaesthetics, intensive care, pain, gastroenterology and radiology specialists, clinical nurse consultant, dietetics, physiotherapy, social work, pharmacy, and clinical psychology (Ansari et al., 2019). Some patients showing high distress levels may be referred to consultation liaison psychiatry for input. Patients are seen preoperatively by the multidisciplinary team in an outpatient clinic and postoperatively by staff in intensive care and on a colorectal ward.

A clinical psychologist provides a psychological assessment consisting of a semi-structured clinical interview and administration of screening tools. The treatment provided is tailored according to individual patient needs based on the outcome of the preoperative psychological assessment. Psychological interventions throughout the patient’s hospital admission might include a combination of the following: psycho-education; emotional validation and distress regulation strategies; motivational interviewing to facilitate treatment compliance and behaviour change; behavioural activation; non-pharmacological strategies for the management of physical symptoms including pain, nausea, and fatigue and delirium; management of emotional problems including anxiety, depression, other psychiatric comorbidities, and fear of cancer recurrence; sleep hygiene strategies; cognitive reframing; supportive counselling to assist with adjustment to significant bodily change; problem-solving strategies to manage barriers to re-adjustment to postoperative life; relationship counselling with consideration of changes to sexuality and reproduction; and consideration of the spiritual aspects of the patient’s suffering, including religious and existential concerns.

The clinical psychologist re-assesses all patients postoperatively to ensure support is provided should there be a change in psychological status. Psychological follow-up after discharge from hospital is also provided for patients on a case-by-case basis. This manuscript focuses on the preoperative and postoperative in-hospital period only.

### Psychological Screening

All patients self-reported symptoms of depression, anxiety, and stress and overall distress levels preoperatively (approximately two weeks before surgery) using the 21-item Depression, Anxiety, and Stress Scale (DASS-21; (Lovibond & Lovibond, 1995) and the 1-item Distress Thermometer (Roth et al., 1998). In addition, the DASS-21 was also administered 10 days postoperatively, whereas both tools (i.e., DASS-21 and Distress Thermometer) were administered on the day of hospital discharge.

The DASS-21 is a validated and reliable tool used to assess psychological symptoms over the previous week in clinical and non-clinical populations (Lovibond & Lovibond, 1995). Several studies have demonstrated favourable psychometric properties of the DASS-21, including in older adults in primary care settings, which demonstrated good-to-excellent internal consistency, very good convergent validity, and acceptable discriminative validity (Gloster et al., 2008). The DASS-21 comprises three 7-item subscales. The 7-item depression scale assesses symptoms including dysphoria, hopelessness, devaluation of life, self-deprecation, anhedonia, inertia and lack of interest. The 7-item anxiety scale assesses the autonomic arousal, skeletal muscle effects, situational anxiety and subjective experience of anxious affects. The 7-item stress scale assesses difficulty in relaxing, nervous arousal, being easily upset or agitated, irritable, over-reacting to situations and impatience. Responses on the DASS-21 are given on a 4-point Likert scale ranging from: 0 = Did not apply to me at all; 1 = Applied to me to some degree, or some of the time; 2 = Applied to me to a considerable degree, or a good part of the time; 3 = Applied to me very much, or most of the time. A subscale score corresponds to levels of symptoms relative to the general population, ranging from 0 (i.e. normal) to 21 (extremely severe). Cronbach’s Alpha for the analysed sample = 0.825.

Distress was measured using the Distress Thermometer (Roth et al., 1998), a sensitive, reliable and valid 1-item self-report measure of psychological distress. The tool has shown to present reasonably psychometric properties within cancer patients (Moretz, 2002; Vodermaier et al., 2009). Patients were asked to rate their distress in the past week on an 11-point visual analogue scale ranging from 0 (no distress) to 10 (extreme distress).

### Surgical Outcomes

The surgical outcomes were derived from a prospective database and included: in-hospital complications (yes/no), completeness of cytoreduction (CC0 = complete[no evidence of disease]/CC1-3 = incomplete[evidence of disease after cytoreduction]) (Jacquet & Sugarbaker, 1996), surgery duration (hours), blood loss (millilitres), length of intensive care unit stay (days), length of hospital stay (days), return to theatre (yes/no), intensive care unit readmission (yes/no), discharge destination (home/ rehabilitation or other hospital), occasions of psychological service (number of occasions between the preoperative period to the day of discharge from hospital).

The Peritoneal Cancer Index is a widely established scoring system that measures the extent of disease (Jacquet & Sugarbaker, 1996). Scores range from 0 to 39, with higher scores indicating more extensive disease.

The Clavien-Dindo classification system was used to determine the severity of postoperative complications (Dindo et al., 2004). Postoperative complications were graded from 0 (no complication) to V (death). For patients presenting more than one postoperative complication, the highest Clavien-Dindo Grade was used. Complications were considered major if they were graded ≥ III.

### Quality of Life

Quality of life outcomes (Ware & Sherbourne, 1992) was measured using the SF-36v2 (Short-Form 36 Version 2) at hospital discharge. This 36-item general quality of life tool is widely used and has great reliability and validity in cancer patients and in cancer survivors.(Reulen et al., 2006; Treanor & Donnelly, 2015) The tool measures two main component summary scores, the physical and the mental component scores. These component scores range from 0 to 100, where a high score indicate better quality of life outcomes.

### Patient Characteristics

These included patient age (years), sex (male/female), primary tumour type (pseudomyxoma peritoneii; appendix adenocarcinoma; colorectal; others) and neoadjuvant (e.g., preoperative) chemotherapy or radiotherapy (yes/no).

### Statistical Analysis

All analyses were performed using SPSS version 28.0.1 (SPSS Inc., Chicago, Illinois). Parametric statistics were used for all analyses. Frequencies (percentages) were used to summarise categorical data, whereas means and standard deviations (SD) were used to summarise continuous data. These descriptive statistics were used to summarise patient characteristics, surgical outcomes, and quality of life. We considered data to be missing at random and no imputation strategy was employed. A limited number of patient characteristics, surgical outcomes, and quality of life were used to avoid Type I error (e.g., approximately one factor per ten patients) (Harrell et al., 1984).

Univariate linear regression analysis was used to evaluate the associations between preoperative DASS-21 subscale scores and Distress Thermometer ratings and patient characteristics, postoperative surgical outcomes, occasions of psychology service, and quality of life scores. Variables with significant univariate associations (*p *< 0.2) were entered into a backwards multivariate regression model. The Bonferroni correction was used in the regression models, with the significance level set to 0.008 for depression, 0.007 for anxiety, 0.01 for stress, and 0.007 for distress (Armstrong, 2014).

The trajectories of DASS-21 subscale scores and Distress Thermometer ratings over the study period were analysed using general linear models with repeated measures. We used a post-hoc paired t-test to explore changes in psychological symptoms over specific time points (e.g., preoperative versus 10 days postoperative). *p *< 0.05 indicates statistical significance.

## Results

### Patient Characteristics and Surgical Outcomes

There were 169 patients who underwent CRS and HIPEC during the study period. The characteristics of the overall sample are presented in Table 1. The majority of patients underwent CRS and HIPEC due to colorectal cancer or appendiceal adenocarcinoma. More than half of the patients had postoperative complications, the most common of which were ileus (paralytic/impaired bowel activity), sepsis, pleural effusion, intraabdominal abscess, and atelectasis (lung collapse). Most patients achieved a complete cytoreduction (CC0). All patients were seen at least once by the clinical psychologist.

**Table 1:** Characteristics, surgical outcomes and quality of life of the included sample

Variables	N (%) or mean ± SD
Age, years	55.3 ± 13.5
Gender, Female	87 (50.3%)
*Tumor Type*	
Pseudomyxoma peritoneii	27 (15.6%)
Appendix adenocarcinoma	35 (20.2%)
Colorectal	79 (45.7%)
Other	32 (18.5)
Neoadjuvant chemotherapy/ radiotherapy	17 (20.2%)
Peritoneal Cancer Index	14.7 ± 10.4
Surgery duration, hours	8.7 ± 2.4
Intensive care unit stay, days	5.7 ± 6.3
Hospital stay, days	20.2 ± 16.5
Return to theatre	15 (8.7%)
Readmission to intensive care	6 (3.5%)
Discharged home	135 (78.0%)
Postoperative complications	107 (61.8%)
Clavien-Dindo Grade I-II	71 (41.0%)
Clavien-Dindo Grade III-V	36 (10.8%)
*Completeness of Cytoreduction (CC)*	
CC0	140 (80.9%)
CC1-3	33 (19.1%)
Blood loss (mL)	1302.1 ± 1017.4
Psychological occasions of service	4.6 ± 3.4
*Quality of Life (Day of hospital discharge)*	
Physical Component Score	35.0 ± 8.9
Mental Component Score	45.4 ± 11.0

Other tumor type include ovarian, peritoneal mesothelioma, primary peritoneal cancer and small bowel adenocarcinoma. Completeness of cytoreduction, CC0 = complete (e.g. no evidence of disease); CC1-3 = incomplete (e.g. evidence of disease after cytoreduction). Quality of life physical and mental component scores range from 0 to 100, higher scores indicate better quality of life

### Depression, Anxiety, Stress, and Distress Pre and Postoperatively

**Fig. 1:**
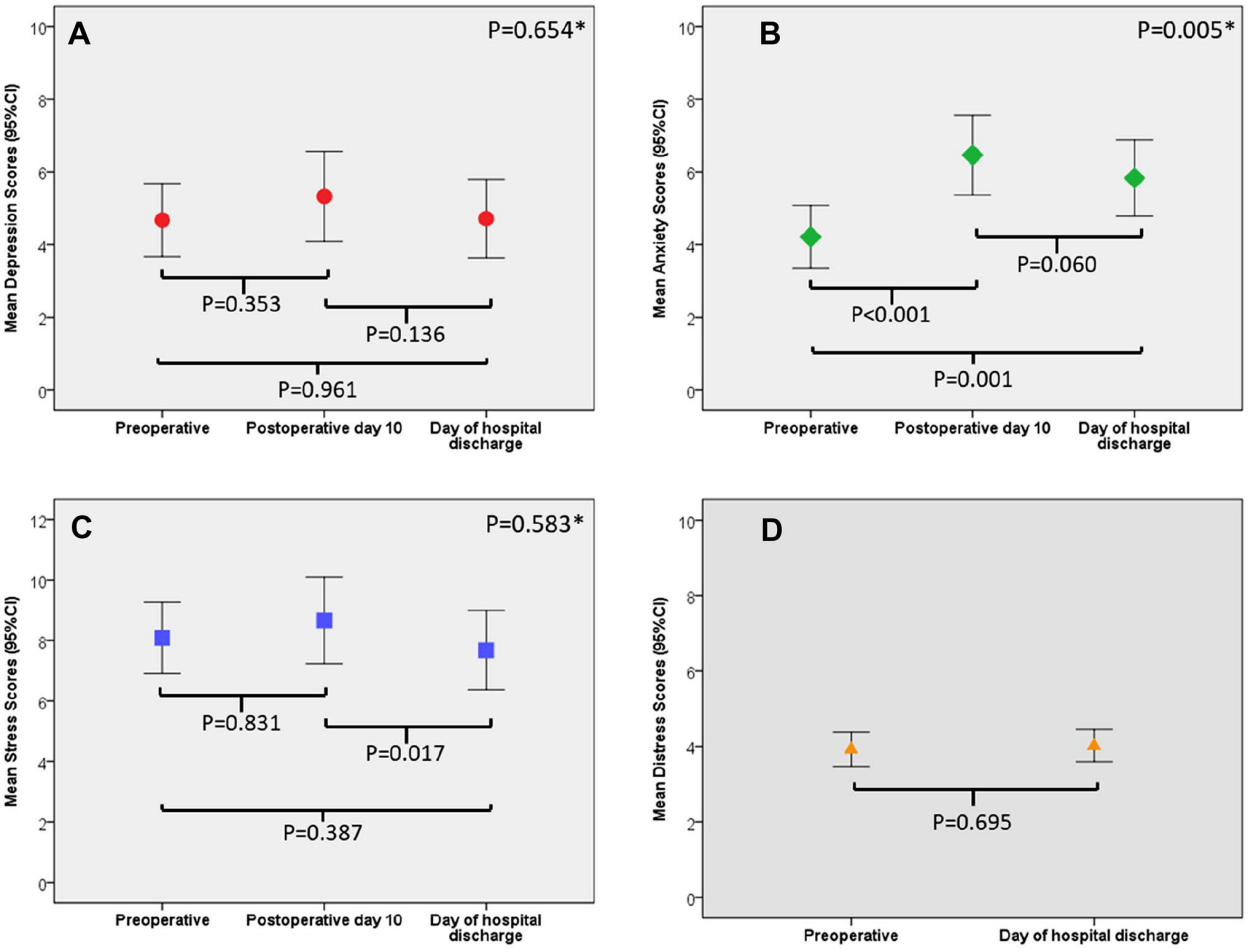
**A–D** Mean depression (**A**), anxiety (**B**), stress (**C**) and distress (**D**) scores following cytoreductive surgery and hyperthermic intraperitoneal chemotherapy. Depression, anxiety and stress measured using the 21-item Depression, Anxiety and Stress Scale (DASS-21) with scores ranging from 0 to 21, whereas a higher score indicated greater symptom severity; distress measures using the distress thermometer, with score ranging from 0 to 10, whereas a higher score indicated greater distress symptom severity; *p *< .05 indicates statistically significancy. *Significance for the overall trajectory

Figure 1A–D shows the mean DASS-21 and Distress Thermometer scores before and after surgery. Preoperative mean DASS-21 subscale scores were 4.7 (SD = 6.6) for depression, 4.2 (SD = 5.7) for anxiety, and 8.4 (SD = 7.8) for stress. The mean preoperative Distress Thermometer rating was 4.0 (SD = 2.9). No changes in DASS-21 depression or stress subscale scores, or Distress Thermometer ratings, were observed from preoperative to day of discharge from hospital. Anxiety scores increased at postoperative day 10 and remained higher than preoperative levels at the time of hospital discharge. The mean DASS-21 stress subscale score decreased from postoperative day 10 to the day of discharge from hospital but was comparable to the preoperative mean.

### Associations Between Preoperative Psychological Variables with Patient Characteristics and Postoperative Outcomes

Tables 2, 3, 4, 5 shows the univariate and multivariate associations between preoperative psychological variables and patient characteristics, surgical outcomes, and quality of life.

**Table 2:** Association between preoperative depression symptoms with patient characteristics, surgical outcomes and quality of life

Variables	Univariate				Multivariate			
	R	R^2^	Unstandardised Coefficient Beta (95%CI)	P Value	R	R^2^	Unstandardised Coefficient Beta (95%CI)	P Value**
Age, years	.189	.036	−0.093 (−0.167 to −0.019)	.014*	.468	.145	–	
Gender, Male	.019	.000	0.253 (−1.768 to 2.274)	.805			–	
*Tumor Type*								
Pseudomyxoma peritoneii	.076	.002	1.385 (−1.408 to 4.177)	.329			–	
Appendix a*denocarcinoma*	.089	.004	1.455 (−1.028 to 3.939)	.249			–	
Colorectal	.169	.032	−2.241 (−4.241 to −0.242)	.028*			–	
Other	.053	.006	0.912 (−1.695 to 3.519)	.491			–	
Neoadjuvant Chemo/radiotherapy, yes	.090	.001	−1.531 (−5.366 to 2.303)	.429			–	
Completeness of cytoreduction, CC0	.085	.007	−1.458 (−4.059 to 1.143)	.270			–	
Surgery duration, hours	.020	.000	0.056 (−0.360 to 0.471)	.792			–	
Intensive care unit stay, days	.041	.002	0.044 (−0.129 to 0.217)	.615			–	
Hospital stay, days	.027	.001	−0.011 (−0.075 to 0.053)	.734			–	
Return to theatre, yes	.077	.002	−1.719 (−5.383 to 1.944)	.355			–	
Readmission to intensive care, yes	.037	.007	−1.263 (−6.881 to 4.356)	.658			–	
Discharged home, yes	.023	.001	−0.538 (−4.484 to 3.407)	.788			–	
Postoperative complications, yes	.040	.002	0.546 (−1.529 to 2.621)	.604			–	
Clavien-Dindo, Grade III-V	.135	.030	−1.948 (−4.763 to 0.868)	.173*			–	
Peritoneal Cancer Index	.125	.028	0.080 (−0.017 to 0.176)	.105*			–	
Blood loss (mL)	.060	.001	0.001 (−0.001 to 0.001)	.439			–	
Psychological occasions of service	.250	.102	0.491 (0.195 to 0.786)	.001*			0.390 (0.030 to 0.750)	.034
*Quality of Life (Day of hospital discharge)*								
Physical Component Score	.034	.001	−0.026 (−0.149 to 0.096)	.974			–	
Mental Component Score	.381	.145	−0.235 (−0.326 to −0.144)	< .001*			−0.182 (−0.302 to −0.062)	.003

Depression measured using the 21-item Depression, Anxiety and Stress Scale (DASS-21) with scores ranging from 0 to 21, whereas a higher score indicates greater depression symptom severity; Physical and mental component scores range from 0 to 100, higher scores indicate better quality of life

*p *< .05 indicates statistical significance

*Variables entered into multivariate analysis

**Bonferroni correction set at *p *= .008

**Table 3:** Association between preoperative anxiety with patient characteristics, surgical outcomes and quality of life

Variables	Univariate				Multivariate			
	R	R^2^	Unstandardised Coefficient Beta (95%CI)	P Value	R	R^2^	Unstandardised Coefficient Beta (95%CI)	P Value**
Age, years	.125	.016	−0.053 (−0.117 to 0.011)	.106*	.450	.138	–	
Gender, Male	.095	.009	−1.077 (−2.800 to 0.646)	.219			–	
*Tumor Type*								
Pseudomyxoma peritoneii	.004	.000	−0.070 (−2.467 to 2.327)	.954			–	
Appendix a*denocarcinoma*	.017	.000	0.236 (−1.898 to 2.370)	.828			–	
Colorectal	.013	.000	−0.153 (−1.889 to 1.584)	.862			–	
Other	.004	.000	0.055 (−2.180 to 2.290)	.961			–	
Neoadjuvant Chemo/radiotherapy, yes	.079	.005	−1.250 (−4.792 to 2.292)	.484			–	
Completeness of cytoreduction, CC0	.147	.022	−2.157 (−4.367 to 0.053)	.056*			–	
Surgery duration, hours	.029	.001	−0.067 (−0.422 to 0.289)	.712			–	
Intensive care unit stay, days	.027	.001	0.025 (−0.126 to 0.175)	.746			–	
Hospital stay, days	.044	.002	−0.015 (−0.070 to 0.039)	.584			–	
Return to theatre, yes	.135	.022	−2.647 (−5.816 to 0.522)	.101*			–	
Readmission to intensive care, yes	.141	.024	−4.216 (−9.062 to 0.630)	.088*			–	
Discharged home, yes	.079	.006	1.636 (−1.785 to 5.058)	.346			–	
Postoperative complications, yes	.043	.002	0.496 (−1.280 to 2.272)	.582			–	
Clavien-Dindo, Grade III-V	.143	.020	−1.807 (−4.262 to 0.648)	.147*			–	
Peritoneal Cancer Index	.015	.000	0.008 (−0.075 to 0.091)	.850			–	
Blood loss (mL)	.009	.000	0.001 (−0.001 to 0.001)	.907			–	
Psychological occasions of service	.173	.030	0.289 (0.033 to 0.545)	.027*			–	
*Quality of Life (Day of hospital discharge)*								
Physical Component Score	.062	.004	−0.041 (−0.145 to 0.063)	.440			–	
Mental Component Score	.316	.100	−0.166 (−0.246 to −0.087)	< .001*			−0.216 (−0.325 to −0.108)	< .001

Anxiety measured using the 21-item Depression, Anxiety and Stress Scale (DASS-21) with scores ranging from 0 to 21, whereas a higher score indicates greater anxiety symptom severity; Physical and mental component scores range from 0 to 100, higher scores indicate better quality of life

*p *< .05 indicates statistical significance

*Variables entered into multivariate analysis

**Bonferroni correction set at *p *= .007

**Table 4:** Association between preoperative stress with patient characteristics, surgical outcomes and quality of life

Variables	Univariate				Multivariate			
	R	R^2^	Unstandardised Coefficient Beta (95%CI)	P Value	R	R^2^	Unstandardised Coefficient Beta (95%CI)	P Value**
Age, years	.162	.026	−0.094 (−0.181 to −0.007)	.035*	.366	.156	–	
Gender, Male	.023	.001	−0.350 (−2.714 to 2.013)	.770			–	
*Tumor Type*								
Pseudomyxoma peritoneii	.063	.004	1.357 (−1.911 to 4.625)	.414			–	
Appendix a*denocarcinoma*	.005	.000	−0.104 (−3.020 to 2.811)	.944			–	
Colorectal	.022	.001	−0.343 (−2.715 to 2.029)	.776			–	
Other	.025	.001	−0.496 (−3.548 to 2.555)	.748			–	
Neoadjuvant Chemo/radiotherapy, yes	.067	.004	1.313 (−3.126 to 5.751)	.558			–	
Completeness of cytoreduction, CC0	.151	.023	−3.025 (−6.042 to −0.007)	.049*			–	
Surgery duration, hours	.019	.000	−0.062 (−0.548 to 0.424)	.802			–	
Intensive care unit stay, days	.064	.004	0.080 (−0.124 to 0.284)	.438			–	
Hospital stay, days	.013	.000	0.006 (−0.068 to 0.080)	.867			–	
Return to theatre, yes	.016	.000	−0.421 (−4.759 to 3.917)	.848			–	
Readmission to intensive care, yes	.001	.000	−0.047 (−6.685 to 6.591)	.989			–	
Discharged home, yes	.016	.000	−0.437 (−5.092 to 4.218)	.853			–	
Postoperative complications, yes	.015	.000	0.235 (−2.194 to 2.663)	.849			–	
Clavien-Dindo, Grade III-V	.071	.005	−1.199 (−4.519 to 2.121)	.475			–	
Peritoneal Cancer Index	.100	.010	−0.075 (−0.188 to 0.038)	.194*			–	
Blood loss (mL)	.016	.000	0.001 (−0.001 to 0.001)	.842			–	
Psychological occasions of service	.204	.041	0.471 (0.120 to 0.822)	.009*			–	
*Quality of Life (Day of hospital discharge)*								
Physical Component Score	.014	.000	0.013 (−0.131 to 0.157)	.860			–	
Mental Component Score	.348	.121	−0.252 (−0.360 to −0.144)	< .001*			−0.267 (−0.376 to −0.158)	< .001

Stress measured using the 21-item Depression, Anxiety and Stress Scale (DASS-21) with scores ranging from 0 to 21, whereas a higher score indicates greater stress symptom severity; Physical and mental component scores range from 0 to 100, higher scores indicate better quality of life

*p *< .05 indicates statistical significance

*Variables entered into multivariate analysis

**Bonferroni correction set at *p *= .001

**Table 5:** Association between preoperative distress with patient characteristics, surgical outcomes and quality of life

Variables	Univariate				Multivariate			
	R	R^2^	Unstandardised Coefficient Beta (95%CI)	P Value	R	R^2^	Unstandardised Coefficient Beta (95%CI)	P Value**
Age, years	.210	.017	−0.046 (−0.079 to −0.013)	.006*	.483	.129	−0.050 (−0.084 to −0.017)	.004
Gender, Male	.122	.005	−0.728 (−1.632 to 0.176)	.114*			–	
*Tumor Type*								
Pseudomyxoma peritoneii	.050	.001	−0.409 (−1.667 to 0.849)	.522			–	
Appendix a*denocarcinoma*	.092	.002	0.675 (−0.441 to 1.792)	.234			–	
Colorectal	.003	.000	0.019 (−0.897 to 0.936)	.967			–	
Other	.054	.001	−0.406 (−1.564 to 0.752)	.490			–	
Neoadjuvant Chemo/radiotherapy, yes	.086	.010	0.682 (−1.069 to 2.433)	.441			–	
Completeness of cytoreduction, CC0	.063	.012	−0.469 (−1.613 to 0.675)	.419			–	
Surgery duration, hours	.034	.000	0.042 (−0.147 to 0.231)	.665			–	
Intensive care unit stay, days	.019	.000	0.009 (−0.068 to 0.086)	.815			–	
Hospital stay, days	.042	.004	0.008 (−0.021 to 0.036)	.594			–	
Return to theatre, yes	.044	.018	0.437 (−1.191 to 2.065)	.597			–	
Readmission to intensive care, yes	.015	.000	0.236 (−2.258 to 2.730)	.852			–	
Discharged home, yes	.125	.018	−1.282 (−2.957 to 0.393)	.132*			–	
Postoperative complications, yes	.022	.000	0.133 (−0.811 to 1.077)	.781			–	
Clavien-Dindo, Grade III-V	.007	.000	0.043 (−1.193 to 1.279)	.945			–	
Peritoneal Cancer Index	.030	.006	−0.009 (−0.052 to 0.035)	.696			–	
Blood loss (mL)	.141	.014	0.001 (0.001 to 0.001)	.068*			–	
Psychological occasions of service	.178	.038	0.155 (0.024 to 0.285)	.021*			–	
*Quality of Life (Day of hospital discharge)*								
Physical Component Score	.074	.002	−0.025 (−0.079 to 0.029)	.355*			–	
Mental Component Score	.408	.100	−0.113 (−0.153 to −0.073)	< .001*			−0.116 (−0.157 to −0.075)	< .001

Distress measured using the Distress Thermometer, with scores ranging from 0 to 10, whereas a higher score indicates greater distress symptom severity; Physical and mental component scores range from 0 to 100, higher scores indicate better quality of life

*p *< .05 indicates statistical significance

*Variables entered into multivariate analysis

**Bonferroni correction set at *p *= .007

In multivariate models, all psychological variables were associated with SF-36v2 mental component summary scores. Lower DASS-21 depression scores were associated with fewer occasions of psychological service (Table 2). No other psychological variables were significantly associated with patient outcomes in multivariate analyses.

## Discussion

This study is the first to our knowledge to report on the course of psychological variables including depression, anxiety, stress and distress in patients within the hospital setting following CRS and HIPEC, and to examine the possible association of these variables with postoperative surgical outcomes. No changes in the reported levels of depression, stress, or distress were observed from the preoperative stage to the day of hospital discharge. Interestingly, the levels of anxiety increased significantly from the initial preoperative measurement to hospital discharge. Most of the postoperative surgical outcomes were not associated with preoperative depression, anxiety, stress or distress. In line with the model of care, patients presenting higher levels of psychological symptoms were seen more frequently by the clinical psychologist and also reported worse mental component quality of life scores at the day of discharge from hospital.

Comparatively and for context, preoperative DASS-21 depression, anxiety, and stress subscale scores in this cohort were similar to normative non-clinical cohorts using the DASS-21 (Ware & Sherbourne, 1992). Similarly, the levels of reported depression and anxiety were in line with the overall levels found in the general Australian population (Lobner et al., 2012). However, this cohort reported much higher levels in the preoperative setting compared to other general population samples using the Distress Thermometer (Fashina et al., 2020). Similar findings were reported in a recent study investigating distress in patients treated with CRS and HIPEC, with a mean preoperative distress rating of 5.4 (Brandl et al., 2019).

Our study highlighted some interesting findings regarding changes to the psychological level of patients during their hospital treatment. The significant increase in anxiety scores observed in the postoperative hospital phase was not surprising given the uncertainty patients face postoperatively, which is driven by a range of considerations. This includes the real threat of complications, adjusting to a stoma, awaiting test results, prolonged recovery, as well as the heavy burden of physical factors including pain, poor sleep, impaired mobility, and malnutrition. A previous study on 305 patients undergoing much less invasive and simpler disc surgery reported similar in-hospital postoperative levels of anxiety, with 23.7% of patients presenting high scores on the Hospital Anxiety and Depression Scale (HADS ≥ 11) (Lobner et al., 2012). Similarly, anxiety levels significantly increased from the preoperative period to one week postoperative in another prospective study involving 37 patients that underwent orofacial cancer surgery (Fashina et al., 2020). In accordance with the model of care described, the CRS and HIPEC patients who reported higher anxiety scores received more occasions of psychological care from the clinical psychologist than those with lower scores. Conversely, we observed no significant changes in the self-reported depression, stress, and distress scores throughout the hospital admission. One possible explanation for this finding is that the interventions provided by the clinical psychologists may have been effective at stabilising the psychological impact of surgery and prevented patient outcomes from worsening. In the current study, as all patients were seen on average 4.6 times by the clinical psychologist from the preoperative period to hospital discharge, the trajectories of their psychological outcome scores without psychological support is not known. The lack of change in the reported scores may have also been because the patients required more time to psychologically adjust to their treatment beyond the in-patient hospital phase and as such prevented meaningful changes in their depression, stress, and distress scores from being detected.

It is important to note that based on the Distress Thermometer, a number of the CRS and HIPEC patients presented a high level of distress both pre- and postoperatively. Although comparable levels of distress were reported in a similar patient cohort with advanced primary or recurrent pelvic cancer using the Distress Thermometer (Young et al., 2014), these levels are concerning and warrant further attention, particularly in the postoperative phase of treatment. There are indications that the provision of information, relaxation techniques, behavioural and cognitive interventions are effective in reducing the level of distress amongst patients scheduled to undergo surgery (Powell et al., 2016) and these, along with other postoperative interventions, will be considered for incorporation into the current model of care.

We also investigated the association between preoperative self-reported depression, anxiety, stress, and distress with patient characteristics, postoperative surgical outcomes, and quality of life. Consistent with research showing that younger age is associated with higher rates of psychological distress and psychiatric problems in adults with cancer (Kroenke et al., 2004; Mor et al., 1994), we found that younger patients tended to report higher levels of stress and distress symptoms. This suggests that younger patients should be assessed at the earliest possible time before surgery to ensure targeted psychological interventions can be implemented pre- and postoperatively.

In terms of postoperative outcomes, the only significant univariate association found was between patients with lower stress levels and completeness of cytoreduction (CC0, *p *= 0.049). However, CC0 was not significant in the multivariate analysis. Our findings differ from the results of a small number of studies in oncology that have suggested a relationship between preoperative psychological status and postoperative surgical outcomes (Hashimoto et al., 2009; Torer et al., 2010). Although limited assumptions can be drawn from this study, it is possible that the psychological support provided within the comprehensive model of care was effective at minimizing the impact of mental ill health on surgical outcomes. Finally, there were significant associations between psychological variables and the mental component scores of the SF-36v2 survey, but this was expected given strong correlations between these measures reported in another cohort of patients (Pfoh et al., 2016).

Whilst this study makes an important contribution to knowledge of the psychological status of CRS and HIPEC patients throughout their in-hospital care, it does have limitations. To the authors’ knowledge, no psychological assessment tools have been validated specifically in patients undergoing CRS and HIPEC, and the measures used in this study may not target all of the relevant issues faced by this population. Additionally, scores on these measures are not a replacement for a clinical diagnosis determined by a mental health professional. Finally, the findings described in this study should be considered within the context of a relatively small cohort of patients, treated at a single centre, without a comparative group. Certainly, it would be beneficial for further research to be undertaken investigating the effectiveness of specific interventions aimed at enhancing the psychological wellbeing of these surgical patients. Although outside the scope of this study, the findings do not contribute to any conclusions about the longer-term psychological and surgical outcomes for these patients. In addition, future research studies should investigate the association between psychological status change over time with patient characteristics, surgical outcomes and quality of life. Other important outcomes that were not explored in this study should also be investigated, including but not limited to marital status, income, educational level and severity of pain. Finally, as previously highlighted, the self-reported assessment of psychological distress from preoperative work-up (approximately two weeks prior to surgery) to hospital discharge may not have provided enough time between these follow-up points for patients to psychologically adjust to their treatment and for a change to be observed.

In conclusion, results from this prospective cohort study demonstrate that most patients scheduled to undergo maximally invasive CRS and HIPEC reported no changes in the clinical course of depression, stress and distress between the preoperative period and hospital discharge, whereas anxiety scores significantly increased during the in-hospital period. Most of the postoperative surgical outcomes investigated were not associated with preoperative levels of depression, anxiety, stress or distress. Whilst the ongoing provision of in-hospital psychological support is critical for this unique patient group, there are opportunities to further develop the psychological measures used, evaluate the effectiveness of specific psychological interventions, and continue developing strategies for reducing the high levels of distress in this population.

## Data Availability

Available upon reasonable request to corresponding author and in accordance with ethics approval.
